# Atopic dermatitis, primary atopic disorders, and the cutaneous microbiome: current understanding of an expanding field

**DOI:** 10.3389/fped.2025.1670623

**Published:** 2025-10-17

**Authors:** Amir Abidov, Diana K. Bayer

**Affiliations:** ^1^ Division of Immunology, University of Iowa Hospitals and Clinics, Iowa City, IA, United States; ^2^ Stead Family Department of Pediatrics, University of Iowa Stead Family Children’s Hospital, Iowa City, IA, United States

**Keywords:** atopic dermatitis, microbiome, primary atopic disorder, immunodeficiency, inborn errors of immunity

## Abstract

Atopic dermatitis is a common inflammatory skin disease with rapidly expanding worldwide prevalence. Increasingly, cases of severe and early-onset dermatitis have been identified and found to be due to underlying monogenic mutations, leading to immune dysregulation. These conditions, called primary atopic disorders, have become an area of extensive study over the last 30 years. Simultaneously, our understanding of the human microbiome has steadily grown, and there is clear evidence that dysbiosis plays a major role in atopic dermatitis, not only in severity of disease and as a potential trigger but also offering clues for targeted treatment strategies. Unfortunately, despite our growing understanding of the cutaneous microbiome and the expanding availability of genetic testing allowing for diagnosis of primary atopic disorders, there remains very limited understanding regarding the microbiomics changes that underlie these disorders. Here we review the current research regarding atopic dermatitis in the setting of primary atopic disorders, understanding regarding primary atopic disorders and associated cutaneous dysbiosis, and identify specific gaps in knowledge.

## Introduction

Atopic dermatitis (AD) is an increasingly common, chronic, inflammatory skin disease characterized by epidermal barrier breakdown and dysregulated inflammation, predominantly via Th2-mediated inflammatory pathways. The resulting pruritic, eczematous lesions are the prototypical early manifestation of the so-called atopic march, the progressive development of AD followed by development of other atopic diseases, such as allergic rhinitis, food allergy, asthma, and eosinophilic esophagitis (1). Recent studies suggest that halting the progression of AD may reduce future systemic allergic sensitization to antigens—although evidence remains limited on the effect this may have on the atopic march (1–4). Given the rising worldwide prevalence of atopic diseases (5), early identification and management of AD has become increasingly critical.

As the focus on AD management has grown, significant progress has been made in understanding the correlation between dysregulation of the skin barrier and changes in the skin microbiome. Enhanced skin colonization by *Staphylococcus aureus* and resultant enzyme and superantigen production has been the best characterized change in the microbiome of patients with AD (6). However, numerous other cutaneous bacterial, fungal, and viral taxa have been identified and studied in the pathogenesis of AD (7). Notably, loss of certain commensal skin bacteria, in particular *S. epidermidis* and *S. hominis,* has also been associated with increased AD severity (8, 9). Recent studies have shown that commensal microbes may have antipathogenic effects via direct pathogen-inhibiting molecules (10–13) and via modulation of the cutaneous barrier (10, 14, 15). Given the microbiome's likely role in the pathogenesis of AD, the effects of specific AD treatments on the skin microbiome have also been studied extensively to better elucidate the pathogenesis of this disease and to develop more targeted treatment options (6). The relationship between the microbiome and skin health is not just skin deep, however, and multiple researcher groups have identified a so-called gut-skin axis, where changes in the gut microbiome may lead to changes in skin health (16–19). These findings imply a microbiome-immune axis, where changes in the human microbiome—regardless of skin location—may lead to increased immune dysregulation.

Given that the changing prevalence of AD cannot be explained by genetic shifts alone (18), there has been an increased interest in the effects of environmental changes leading to a propensity for AD development (18, 20). The list of environmental factors affecting AD development is vast and includes pollutants, rural vs. urban living, allergens, medications, and microbial exposures (including to antibiotic-resistant pathogens) (18, 21, 22). In recent decades, cases of very early onset, severe, and unique presentations of AD have also been identified. These cases have led to the characterization of a group of inborn errors of immunity typically presenting with early and severe AD, termed primary atopic disorders (PADs) (23). PADs are defined as monogenic diseases presenting with significant allergy and/or atopy as characteristic features, frequently manifesting with an eczematous dermatitis (23). Most PADs have been characterized within the last 30 years (24). Apart from highly prevalent loss-of-function (LOF) variants in *FLG*, which codes for the crucial epidermal barrier protein filaggrin, most PADs result in significant immune dysfunction with high risk for severe infections (23–26). Categorization of these disorders is not standardized given significant functional and symptomatic overlap, and new PADs are rapidly being discovered. Additionally, it appears likely that environmental exposures may further modulate clinical onset of PADs (22, 27), leading to variability in presentations. Given the importance of early treatment of these immune compromised patients, early diagnosis is paramount.

While the skin microbiome in AD has been extensively researched, there is very limited available literature regarding differences in the skin microbiome of patients with PADs. This is in contrast to the gut microbiome in primary immunodeficiencies, which has been evaluated in much greater detail (17, 28). Treatments modifying the gut microbiome in patients with PADs have also been studied (17, 28, 29).

**Figure 1:**
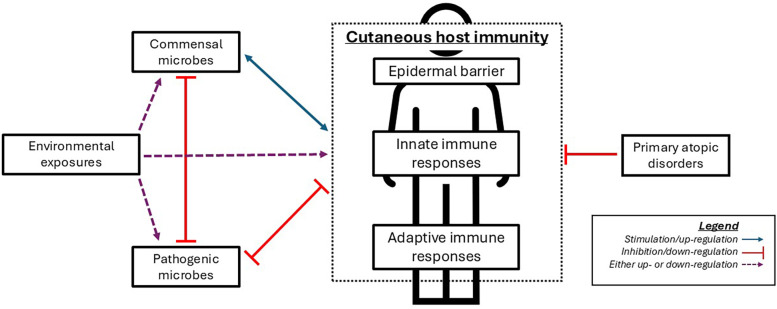
Simplified representation of interactions between microbes, cutaneous immunity, and primary atopic disorders. Commensal microbes play a major role in cutaneous immune function via inhibition of potential pathogens and regulation of certain immune functions. Appropriate immune responses help foster a healthy microbiome, which conversely fosters appropriate immune responses. Disruption in normal cutaneous immunity (e.g., via environmental exposures or primary atopic disorders) leads to microbiome changes which promote pathogenic microbes, which can further inhibit the normal microbiome and normal immune function.

The available data reviewed in the following sections suggests that immune dysfunction in PADs significantly influences the cutaneous microbiome. In Figure 1, we review the factors that influence AD and associated cutaneous microbiome alterations, including in this unique patient population. Later, we will discuss the current understanding of the difference in the skin microbiome in patients with the most extensively studied PADs, along with a general review of these PADs and of diseases that mimic PAD pathology.

## Skin microbiome in AD

We have been aware in recent years of the important role that commensal microorganisms play in normal immune function. The healthy skin microbiome consists of a diverse community of bacteria, fungi, and viruses, responsible for impeding the growth of pathogens, presumably through competition and both direct and indirect antimicrobial effects. Bacteria make up the majority of commensal microbes, with *Corynebacterium, Cutibacterium, Micrococcus, Staphylococcus, Streptococcus,* Betaproteobacteria, and Gammaproteobacteria being the most common (30). *Malassezia* spp., in particular *M. globosa* and *M. restricta*, are the most prevalent fungal colonizers (31)*.* Skin microbial diversity varies by body location, the type of skin, skin moisture, patient age, and patient ethnicity, with healthy children displaying especially diverse microbiota as compared to adults (6, 30).

In patients with AD, skin microbial composition differs compared to controls with over-representation predominantly of *S. aureus*, although a definitive causal relationship has not been clearly defined. In disease flares, diversity appears to shift towards less varied communities with an increased proportion of *S. aureus* in the skin compared to after flares are resolved; a similar difference is noted between lesional and non-lesional skin in patients with AD (30, 32). Conversely, increased proportions of certain *Staphylococcus* species (such as *S. epidermidis* and *S. hominis*) and other common commensal bacteria (*Streptococcus*, *Corynebacteria,* and *Propionibacterium*) have been associated with reduced AD rates and severity (33). Many of these commensal organisms have been found to directly and indirectly protect host skin via multiple mechanisms, such as secretion of lantibiotics or promoting antimicrobial peptides such as β-defensins which may suppress *S. aureus* (6, 9). In patients with AD, the majority of *S. aureus* strains produce superantigens, such as staphylococcal enterotoxin B, which can further exaggerate Th2 inflammatory responses and exacerbate AD (2, 9, 34). Other studies have focused on differences in fungal communities (31, 35), noting relative enrichment of certain *Malassezia* spp. (*M. dermatis, M. sloofiae*, and *M. sympodalis*) in AD. These studies have been difficult to consistently replicate due to variance between lesional and non-lesional skin, differences in skin sampling sites, microbial changes with patient age, and differences in disease activity; all of these factors influence the skin microbiome (30). In addition, evidence suggests that there are differences in virulence factors between certain strains of *S. aureus*, with those found on active AD lesions inducing skin inflammation in mouse models, more-so than *S. aureus* from healthy human skin (34).

Lastly, environmental exposures have a major effect on the skin microbiome, affecting microbial diversity and quantity (22, 36–38). The environmental factors affecting the skin microbiome are similar to those associated with AD, such as medication exposures, rural vs. urban environments, climate changes, pollutants, and allergens. Specifically, these environmental exposures appear to greatly affect the abundance of pathogenic microbes (in particular, *S. aureus* and pathogenic fungi), and changes in commensal microbial taxa (22, 38).

### Effects of treatments on the skin microbiome

With advances in skin microbiome research, we have begun to understand the effects that targeted therapeutic strategies may have on the skin microbiome. Numerous studies have evaluated the effects of treatments in patients with AD on the skin microbiome and *S. aureus* in particular, excellently summarized by Demessant-Flavigny et al. and Huang et al. (6, 39). As a whole, multiple studies evaluating both indirect (emollients, anti-inflammatory topicals, monoclonal antibodies) and direct antibacterials (including antiseptics, topical and systemic antibiotics, and *S. aureus-*specific therapies including anti-*S. aureus* endolysin and bacteriotherapy) have shown beneficial changes in *S. aureus* populations and increases in commensal bacteria (6, 33, 39–44). Of the monoclonal antibodies approved for AD treatment, the microbiome-modulating effects of the interleukin (IL)-4 receptor alpha antagonist dupilumab and IL-13 antagonist tralokinumab have both been evaluated (43–46). Of the three studies evaluating dupilumab and one study evaluating tralokinumab, all excluded pediatric patients, and all showed improvement in cutaneous dysbiosis, reduction in *S. aureus* abundance, and increases in *S. epidermidis* and *S. hominis.* While Janus Kinase (JAK) inhibitors were recently approved for treatment of AD, there is thus far limited understanding of the effects these therapies may have on the skin microbiome (47).

Cutaneous probiotics (live microbes) and direct cutaneous microbial transplantation has been explored, with variable efficacy in clinical studies (9, 21, 39). However, the use of postbiotic therapies (beneficial non-live metabolic byproducts of probiotic microbes) has shown promising results in clinical studies with lower theoretical risk than probiotics (48), which may be a concern in patients with certain PADs. Notably, a number of trials using oral probiotics have shown improvement in AD with treatment (16), further solidifying the gut-skin axis.

## Primary atopic disorders

As noted previously, PADs encompass a large group of monogenic defects leading to significant allergic and/or atopic diseases, with eczematous dermatitis as a common presenting feature. To date, there have been at least 48 single-gene defects identified as PADs, most of which are associated with underlying immune dysregulation (23). Many PADs can have catastrophic implications for patients, frequently requiring early and aggressive treatment, including potential hematopoietic stem cell transplantation, making early identification and expanded treatment strategies increasingly important. Standardized categorizations of PADs have not been established, although certain groupings are commonly used (Table 1). Very broadly, the immune dysregulation of these disorders leads to variable combinations of: (1) propensity for Th2 pathways, either via direct upregulation or loss of downregulation (2) dysfunctional T-regulatory (Treg) cell pathways, leading to loss of self-tolerance and (3) direct loss of epidermal barrier function (49).

**Table 1:** Select PADs with dermatitis as a characteristic feature.

Disease or syndrome name	Gene	Clinical features
Filaggrin deficiency	*FLG*	Severe AD, ↑ IgE
Hyperimmunoglobulin E Syndromes (HIES) and Similar Clinical Phenotypes		
Autosomal dominant-HIES/STAT3-HIES	*STAT3 (LOF)*	Severe dermatitis, ↑ IgE, eosinophilia, recurrent skin abscesses, CMC, recurrent pneumonia, bone fragility, scoliosis, joint hyperextensibility, retained primary teeth, dysmorphic facial features
AR-HIES/DIDS	*DOCK8*	Severe AD, ↑ IgE, eosinophilia, food allergy, CMC, cutaneous infections (esp. molluscum, papilloma virus, herpes simplex), malignancy, autoimmunity
Variant STAT3-HIES/AR GP130 deficiency	*IL6ST*	Similar to STAT3-HIES, destructive lung disease, +/- neurodevelopmental delay
Variant STAT3-HIES/AR IL-6 receptor deficiency	*IL6R*	Similar to STAT3-HIES, typically without skeletal abnormalities
Variant STAT3-HIES/HIES3	*ZNF341*	Similar to STAT3-HIES
ERBIN deficiency	*ERBB2IP*	Similar to STAT3-HIES
STAT5b deficiency	*STAT5b (LOF)*	↑ IgE, postnatal growth impairment, growth hormone insensitivity Can have IPEX-like presentation
STAT6 gain-of-function	*STAT6 (GOF)*	↑IgE, severe atopy, ↑ risk for hematologic malignancy
TYK2 deficiency	*TYK2*	Similar to STAT3-HIES in some cases; ↑ susceptibility to intracellular bacteria (mycobacteria), viral infection
PGM3 deficiency	*PGM3*	↑IgE, severe atopy, ↑ rate of bone marrow failure, skeletal dysplasia, neurodevelopmental delay
Wiskott-Aldrich syndrome (WAS) and Similar Clinical Phenotypes		
WAS	*WAS*	Severe AD, thrombocytopenia with small platelets, recurrent infections (bacterial, viral), hematologic malignancy, autoimmunity, bloody diarrhea
WAS 2/WIP deficiency	*WIPF1*	Severe AD, thrombocytopenia with small platelets, recurrent infections (bacterial, viral), bloody diarrhea
ARPC1B deficiency	*ARPC1B*	Similar to WAS, milder
CBM complex-associated diseases		
CADINS	*CARD11*	General atopy, ↑ IgE, eosinophilia, respiratory and cutaneous viral infections
CARD14 deficiency	*CARD14*	General atopy, recurrent respiratory and cutaneous pyogenic and viral infections
MALT1 deficiency	*MALT1*	Similar to CADINS with ↑ risk of IBD
Additional PADs		
Netherton syndrome	*SPINK5*	Congenital ichthyosis, “bamboo hair”, ↑ IgE, ↑ risk of enteropathy, failure to thrive
IPEX syndrome	*FOXP3*	Severe eczematous dermatitis, ↑ IgE, ↑ IgA, recurrent severe infections, autoimmune enteropathy, polyendocrinopathy
RLTPR deficiency	*CARMIL2*	General atopy, recurrent respiratory and cutaneous infections, malignancy, and EBV-associated lymphoproliferative disease
Severe Combined Immunodeficiency (SCID) Phenotypes		
Omenn syndrome	Multiple genes: *RAG1, RAG2, IL7RA, ZAP70, ADA, DCLRE1c, RMRP, CHD7*	Very early onset eczematous dermatitis (<2 months), erythroderma, combined immunodeficiency, eosinophilia

Other clinical features and causative genes are summarized here. PADs are grouped by their general clinical features and diseases they may mimic. PADs that do not cause dermatitis as a prominent feature are not included here. PAD, primary atopic disorder; AD, atopic dermatitis; AR, autosomal recessive; LOF, loss of function; GOF, gain of function; IL, interleukin; CMC, chronic mucocutaneous candidiasis; WIP, WAS/WASL interacting protein; IPEX, immunodysregulation polyendocrinopathy enteropathy X-linked; CBM, caspase recruitment domain (CARD) proteins, B-cell CLL/Lymphoma 10 (BCL20), and mucosa-associated lymphoid tissue lymphoma translocation protein 1 paracaspase (MALT1); CADINS, CARD11-associated atopy with dominant interference of NF-κB signaling; EBV, Epstein–Barr virus.

In the following sections, we will discuss the most studied PADs to date, their clinical presentations, distinguishing features, associated dermatologic findings, and current understanding of their effects on the host cutaneous microbiome (Table 2). When available, we will review PAD-specific AD treatment evidence in the respective disorder section. A detailed review of the current knowledge regarding gut microbiome changes in patients with PADs, among other inborn errors of immunity, has been recently published by Hazime et al. (17) and will not be reviewed in detail here.

**Table 2:** Summary of PADs with available cutaneous microbiome data.

Disease	Gene	Skin microbiome	Characteristic cutaneous infections
Normal skin (6, 55)	Wild type	• Wide diversity • Uncommon colonization with *S. aureus* (10%–20%)	
Atopic dermatitis (6, 9, 55)	Wild type	• ↑ *S. aureus,* certain *Malassezia* spp.*,* and *Candida* colonization • ↓ common commensal microbiomes, including other *Staphylococcus* spp.	• *S. aureus*, *Candida*
Filaggrin deficiency (6, 55)	*FLG*	• ↑ *S. aureus,* certain *Malassezia* spp.*,* and *Candida* colonization • ↑ *S. aureus* biofilm propensity, pathogenicity • Non-lesional skin is similar to lesional skin of patients with AD	• *S. aureus*, *Candida*
STAT3-HIES (63–65)	*STAT3*	• Colonization by *Serratia marcescens*, *S. aureus*, *Corynebacterium* spp., *Candida* spp., and *Aspergillus* spp. • *S. aureus* strains display ↑ virulence genes and antibiotic resistance • *S. aureus* and *S. haemolyticus* enriched	• Recurrent “cold” abscesses associated with *S. aureus*, *Candida* (CMC) • Cutaneous viral infections are less common than in DIDS
DIDS (63, 68, 70)	*DOCK8*	• Similar to STAT3-HIES, with ↑ viral colonization (*Papillomaviridae, Polyomaviridae,* and *Poxviridae* predominance) • Limited data on bacterial populations	• Cutaneous viral infections, especially MC, HSV, and HPV • Otherwise, similar to STAT3-HIES
Wiskott-Aldrich syndrome (63)	*WAS*	• Limited data in eczematous patients • ↑ bacterial community diversity (retroauricular crease only)	• Cutaneous viral infections, bacterial cellulitis and abscesses, *S. aureus* predominant
Netherton syndrome (87)	*SPINK5*	• ↓ microbial diversity • ↑ in *S. aureus, S. epidermidis, Strep agalactiae* • ↑ *S. aureus* bacterial virulence peptides and proteases (PSM*α*, Staphopain A and B)	• Cutaneous bacterial infections, gastrointestinal infections, rare invasive infections

Summary of PADs with data available regarding cutaneous microbiome changes, compared to wild type controls. PADs without available literature were not included. LOF, loss of function; HIES, hyperimmunoglobulin E syndrome; AD, atopic dermatitis; CMC, chronic mucocutaneous candidiasis; DIDS, DOCK8 immunodeficiency syndrome; MC, molluscum contagiosum; HSV, herpes simplex virus; VZV, varicella zoster virus; HPV, human papillomavirus; PSMα, phenol-soluble modulin alpha.

### Filaggrin deficiency

LOF variants of the gene *FLG*, which encodes filament aggregating protein (filaggrin)*,* cause the most common PAD (50, 51). *FLG* LOF mutations with variable degrees of function follow a semi-dominant inheritance pattern, with homozygous or compound heterozygous genotypes conferring increased risk of AD and an early presentation of AD (within the first months of infancy) (52, 53). While *FLG* LOF is not specifically associated with immune deficiency, skin barrier breakdown in these patients can lead to increased cutaneous infections and immune dysregulation. The AD affecting these patients may also be treatment-resistant. Although filaggrin deficiency is the most common PAD, the availability of diagnostic genetic testing is limited due to challenges of sequencing this gene (54).

Patients with filaggrin deficiency have underlying changes in their cutaneous microbiome—notably, an increased prevalence of *S. aureus* and *Malassezia* colonization, with overall reduced microbial diversity compared to wild type controls (7, 55). Patients with filaggrin deficiency may have a predilection for more pathogenic *S. aureus* strains with higher biofilm forming propensity (6, 55). In addition, there may be less lesional vs. non-lesional skin divergence in these patients, and earlier onset of dysbiosis (7, 47, 55).

### Hyperimmunoglobulin E syndromes

Hyperimmunoglobulin E syndromes (HIES) were originally defined as two primary variants, each with mutations in a different gene: an autosomal dominant variant caused by loss of function of the signal transducer and activator of transcription (STAT) 3 gene, *STAT3*, and an autosomal recessive variant due to loss of function of the dedicator of cytokinesis 8 gene, *DOCK8*. Over time, numerous genotypes with similar clinical phenotypes have been identified; HIES has thus become somewhat of a misnomer as many PADs may present with very elevated IgE levels (25, 26). For example, filaggrin deficiency, which is not commonly considered an inborn error of immunity, is also associated with high levels of IgE due to AD (25, 26). Thus, while we will use the term HIES here to define a set of diseases characterized by very elevated IgE levels, elevated IgE levels can be seen in many patients with AD without an overt PAD due to many factors, including but not limited to the increased Th2 skew associated with AD and induction of IgE production by environmental factors such as *S. aureus* colonization (18, 56).

### STAT3-HIES

The most common form of HIES continues to be dominant-negative *STAT3* (STAT3-HIES, or autosomal dominant HIES) mutations, previously called “Job's Syndrome”. STAT3 plays a key role in the differentiation of Th17 cells, with downstream downregulation of Th2 pathways (57). This disease is characterized predominantly by elevated IgE, eosinophilia, severe eczematous dermatitis as early as the first month of life, recurrent skin abscesses without the typical inflammatory signs (warmth, erythema, or tenderness; “cold abscesses”), recurrent cyst-forming pneumonias, and chronic mucocutaneous candidiasis (CMC) (25, 26). The eczematous dermatitis of STAT3-HIES tends to be severe and does not necessarily meet strict clinical criteria for AD (25, 58, 59). While STAT3-HIES-associated dermatitis is generally treatment-resistant, dupilumab appears to be effective in treating dermatitis in these patients (60–62).

Other atopic features are less common in patients with STAT3-HIES compared to wild-type patients with AD (59). Multiple extracutaneous findings, including retained primary teeth, minimally traumatic bone fractures, characteristic facial features, and scoliosis, may be present later in life (25).

STAT3-HIES appears to affect the cutaneous microbiome (63–65). In general, the skin of these patients shows decreased microbial diversity, loss of some commensal strains, and increase in certain pathogenic bacterial and fungal strains (63–65). The strains of *S. aureus* affecting these patients tend to be more likely to express methicillin resistance, Panton-Valentine Leukocidin (PVL), and staphylococcal enterotoxins K and Q (SEK and SEQ, respectively) (60, 61, 64). PVL is a pore-forming cytotoxin associated with methicillin resistance, while SEK and SEQ are non-classical staphylococcal superantigens rarely expressed in wild-type patients with AD (64). While overall *S. aureus* presence was not increased in most patients—likely due to widespread use of *S. aureus*-targeting therapies—the strains present did appear more pathogenic. Other *Staphylococcus* species, including *S. epidermidis* and *S. haemolyticus*, were enriched in these patients. Fungal colonization with relatively increased *Candida* and *Aspergillus* spp. abundance was noted, likely due to the deficiency of Th17 cells observed in STAT3-HIES. Interestingly, these patients were noted to have novel skin colonization with *Serratia* species (specifically *S. marcescens*), with increased variance between patients with STAT3-HIES compared to controls. In addition to *Serratia* species, *Acinetobacter* species also seem to have an increased prevalence in these patients, while commensal *Corynebacterium* spp. were less prevalent, loss of which may further inhibit host immune responses to *Candida* spp. and *S. aureus* (60, 61, 65).

### DOCK8 deficiency

LOF mutations in *DOCK8* are the next most common HIES and follow an autosomal recessive pattern, often termed DOCK8 immunodeficiency syndrome (DIDS) or autosomal recessive HIES. We will use DIDS to distinguish it from other autosomal recessive HIES variants. Patients with DIDS have markedly impaired T-cell differentiation and function, leading to significant immune dysregulation (26, 66).

Like STAT3-HIES, patients with DIDS have the classic features of high IgE, eosinophilia, severe AD, skin infections (abscess), and CMC, but are distinguished by an increased propensity for cutaneous viral infections and increased risk for autoimmunity and malignancy (67). These cutaneous viral infections include infections with molluscum contagiosum (MC), herpes simplex virus (HSV), and human papillomaviruses (HPV) and may be treatment-resistant (25, 66, 68).

Additionally, DIDS-associated eczematous dermatitis is more consistent with typical AD compared to the eczematous dermatitis of STAT3-HIES (26, 59, 66). Musculoskeletal and dental abnormalities are rare as compared to STAT3-HIES (25, 26). The increased malignancies observed are primarily lymphomas and cutaneous squamous cell carcinomas (59).

DIDS-associated cutaneous dysbiosis has been analyzed in multiple studies. Generally, the bacterial pathogens are similar to those found in patients with STAT3-HIES (63), with a notable difference in the cutaneous virome (63, 68). Patients with DIDS have profoundly elevated relative abundances of certain eukaryotic viruses in the skin, with *Papillomaviridae, Polyomaviridae,* and *Poxviridae* being the most predominant (68). This is consistent with the typical clinical features of resistant cutaneous infections with MC and HPV in these patients.

Similar to STAT3-HIES, patients with DIDS frequently have treatment-resistant AD, and the efficacy of dupilumab in this population has been described in limited case reports demonstrating efficacy of dupilumab treatment (61, 62, 69). Notably, dupilumab appears to benefit both the AD and reduce skin infections in these patients. More recently, Che et al. followed 24 patients with DIDS through hematopoietic stem cell transplantation (HSCT), showing that HSCT had dramatic effects not only on the cutaneous microbiome of these patients, but functionally resolved the skin disease of many of these patients (70). These patients showed normalization of their skin microbiomes closer to healthy controls, regaining site-specific patterns, and dramatic reductions in *S. aureus* and viral abundance.

### STAT3-HIES phenocopies

Mutations in other genes can present phenotypically like STAT3-HIES, as these variants affect proteins crucial to the STAT3 signaling pathway. Normal IL-6 signaling is transduced in large part via STAT3. Autosomal recessive variants of the IL-6 receptor gene, *IL6R*, present similarly to STAT3-HIES but lack the skeletal abnormalities (25, 26, 71). Variants of the IL-6 Cytokine Family Signal Transducer gene, *IL6ST*, which has both autosomal dominant and autosomal recessive LOF variants, have phenotypes that resemble that of STAT3-HIES but are associated with neurodevelopmental delay, destructive lung disease, and bronchiectasis (72, 73). *ZNF341* (zinc finger protein 341) encodes a transcription factor involved in the STAT3 signaling pathway; LOF variants of *ZNF341* cause a syndrome phenotypically identical to STAT3-HIES by impacting DNA binding by ZNF341 (26). Finally, individuals with ERBIN deficiency due to autosomal dominant *ERBB2IP* LOF present very similarly to patients with STAT3-HIES but with fewer infections. The protein ERBIN forms a complex with STAT3 to facilitate STAT3 signaling (23, 24, 26).

### Other variants of HIES

Mutations of other STAT and STAT-related genes have also been implicated in early childhood dermatitis and elevated IgE, including LOF mutations of *STAT5b* and gain-of-function (GOF) mutations of *STAT6* (23, 24, 74). STAT5b is required for the response of naïve T cells to IL-2, triggering production of the IL-4R*α* subunit (75), and STAT6 is required for differentiation of Th2 cells (74, 75). Notably, *STAT5b* LOF mutations are associated with a unique phenotype of postnatal growth impairment due to growth hormone insensitivity. Autosomal recessive TYK2 deficiency has also been described with a HIES-like clinical phenotype in some affected patients, associated with increased susceptibility to viral, intracellular bacterial, and mycobacterial infections (25, 76).

Autosomal recessive hypomorphic mutations in the phosphoglucomutase 3 gene *PGM3* can lead to a clinical SCID phenotype with features of HIES, with elevated IgE, severe atopy, systemic bacterial infections, disseminated Herpesvirus infections, neurologic impairment, and increased autoimmunity (25).

### Wiskott-Aldrich syndrome and similar syndromes

Mutations in the Wiskott-Aldrich syndrome gene, *WAS*, which codes for WAS protein (WASp), can lead to an eponymous X-linked immunodeficiency called Wiskott-Aldrich syndrome (WAS) (26, 77). WASp is a key protein in the signal transduction and actin polymerization pathways of hematopoietic cells, and certain variants can lead to combined immune deficiency, thrombocytopenia with small platelets, and eczematous dermatitis, often within the first month of life (77). The eczematous dermatitis of WAS affects the majority of patients and generally meets clinical criteria for AD but can be abnormally severe, widespread, and often difficult to treat (59). Along with AD, complications of thrombocytopenia are often one of the first clinical presenting features (25, 26, 67, 77).

Patients with WAS may have aberrant regulatory T cell (Treg) function, which is likely largely responsible for the increased rate of autoimmunity in this population (77, 78). There is a notably increased rate of hematologic malignancy as well. Other mutations in *WAS* may lead to less severe phenotypes, such as X-linked thrombocytopenia, which lack infectious and dermatologic complications (77).

There is very little known regarding changes in the microbiome of patients with WAS. The only available study on skin microbiome dysbiosis in humans to date (63) included patients that did not have the severe eczematous phenotype, with significantly lower SCORAD (Scoring Atopic Dermatitis) scores and with lower IgE levels than included patients with AD, STAT3-HIES, and DIDS. These patients had microbial colonization generally more similar to healthy controls than to those of other PADs (specifically, STAT3-HIES or DOCK8 deficiency), suggesting the possibility of confounding due to the difference in their specific disease phenotype. However, a mouse model of WAS (79) did note significant dysbiosis with a relative abundance of certain genera (*Streptococcus* and *Helicobacter*) and novel colonization not detected in wild-type mice. Some of these changes began as early as the first week of life. Fortunately, treatment of WAS with both gene therapy and hematopoietic stem cell therapy have been reported to be effective in improving AD in these patients (80, 81).

Multiple other PADs may present similarly to WAS without WASp deficiency. Loss of function variants of *WIPF1* (WAS/WASL interacting protein family member 1) can lead to an autosomal recessive variant of WAS called WAS 2, with a similar clinical presentation (23, 75). A somewhat milder variant of a WAS-like syndrome may also present secondary to *ARPC1B* LOF, with more mild thrombocytopenia but otherwise similar clinical phenotype (26).

### CBM complex-associated disorders

Caspase recruitment domain (CARD) proteins, B-cell CLL/Lymphoma 10 (BCL10), and mucosa-associated lymphoid tissue lymphoma translocation protein 1 paracaspase (MALT1), interact to form what is known as the CARD-BCL10-MALT1 (CBM) complex (25, 75, 82). The CBM complex regulates activation of NF-*κ*B pathways, facilitating T cell receptor signal transduction, loss of which leads to the Th2 phenotype. Mutations in the genes encoding these proteins lead to so-called “CBM-opathies” (25).

*CARD11* and *CARD14* dominant-negative mutations can both lead to severe atopy, recurrent viral respiratory and cutaneous infections, with *CARD11* showing a more Th2-skewed immune response (25, 26, 82). Patients with *CARD11* LOF frequently have treatment-resistant AD, although both dupilumab and omalizumab have been reported to be effective as treatments (83).

*MALT1* LOF has a similar phenotype, with an increase in gastrointestinal infections and loss of self-tolerance, predisposing to inflammatory bowel disease (25, 26). Use of hematopoietic stem cell transplant has been reported to also treat the AD of patients with *MALT1* LOF (84, 85).

### Netherton syndrome

Mutations in the serine protease inhibitor Kazal type 4 gene (*SPINK5*) lead to a loss of function of the protein lymphoepithelial Kazal-type-related protease inhibitor (LEKTI-1) (86). Loss of LEKTI-1 leads to increased protease activity, thereby increasing skin barrier damage and epidermal inflammation. This monogenic, autosomal recessive disease is called Netherton syndrome or Comèl-Netherton syndrome and is characterized by congenital ichthyosiform erythroderma and severe eczematous dermatitis, classic hair shaft abnormalities (trichorrhexis invaginata or “bamboo hair”), potential failure to thrive, and the development of significant atopic disease. Skin infections in this population are very common (86).

The lesional skin in patients with Netherton syndrome is dominated by *S. aureus* and *S. epidermidis*, isolates of which are both able to promote skin inflammation in mouse models (87). The secreted virulence peptides and proteases of these *S. aureus* isolates have also been associated with an increased frequency of childhood skin infections (87). Notably, patients with Netherton syndrome do not seem to have severe underlying systemic immune deficiency, meaning their immune dysregulation and recurrent skin infections are more likely to be related to severe barrier dysfunction (88).

### Other monogenic disorders

Immune dysregulation polyendocrinopathy enteropathy X-linked (IPEX) syndrome, caused by LOF of *FOXP3*, leads to significant Treg dysfunction. This leads to a PAD characterized by elevated IgE levels, eczema, eosinophilia, autoimmune enteropathy, autoimmune endocrinopathies, and severe infections (25, 26, 59). Diseases with IPEX syndrome-like presentations include CD25 deficiency, which is autosomal recessive with chronic viral, fungal, and bacterial infections, and the previously reviewed STAT5b deficiency, distinguished by growth-hormone insensitive dwarfism (25, 26).

RLTPR deficiency, caused by autosomal recessive mutations of *CARMIL2*, leads to an atopic phenotype characterized by recurrent infections, malignancy, and Epstein–Barr virus-associated lymphoproliferative disease (25, 26).

### Severe combined immunodeficiency (SCID) and similar presentations

Many patients with SCID and SCID-like diseases may present early in life with severe eczematous dermatitis, severe immunodeficiency, and autoimmunity. These are features of Omenn syndrome (most commonly due to mutations in *RAG1* or *RAG2*) and more mildly of adenosine deaminase severe combined immunodeficiency (ADA-SCID) (26, 89). However, this presentation may be seen with other SCID genotypes, including mutations in *IL7RA, ZAP70, IL2RA*, *DCLRE1C, RMRP*, and severe pathogenic variants of *CHD7* (25, 26, 89). Many of these patients, particularly those with Omenn syndrome, have early onset eczematous dermatitis, presenting as early as birth. These patients will frequently present with dermatitis that does not technically meet classification criteria for AD and is often treatment-resistant (25, 26).

The skin and gut microbiome in patients with hypomorphic *RAG* mutations has been described in detail by Blaustein et al., although none of these patients were reported to have severe eczematous dermatitis as can be seen in patients with Omenn syndrome (90). Regardless, this study showed significant changes in baseline gut and skin microbiomes compared to healthy controls with loss of body site specificity, increased inter-individual variation, and colonization with microbes (including bacteria, fungi, and viruses) not previously described on human skin.

## Discussion and conclusions

Early presentation of severe atopy, often presenting as severe eczematous dermatitis, is a clear warning sign for underlying immune dysregulation and should raise concern for underlying immune deficiencies or PAD. While our understanding of the existence and clinical importance of PADs has grown, significant knowledge gaps regarding PADs persist.

Even in patients without PAD, AD is a complex disease caused by the interaction of immune dysfunction, skin barrier disruption, and microbiome changes, and is highly associated with increased risk for future atopic diseases. Our understanding of the effects of microbiome-immune system crosstalk has rapidly expanded in recent years, especially in the context of atopy. Despite our improved understanding of the alterations in the microbiome in patients with AD, little is known regarding cutaneous microbiomes in patients with PADs, despite the growing recognition of PADs as a group. This knowledge gap affects both patients with and without PADs—the specific immune dysfunction highlighted by each PAD provides clinicians with important information regarding the specific roles of individual components of cutaneous immunity. Understanding which unique pathogens affect patients with specific PADs may further unlock understanding of the virulence factors these pathogens may produce and the importance of certain commensal microbes in the human cutaneous microbiome. In the future, this research may unlock avenues of treatment for patients with and without PAD, with the eventual goal of preventing AD onset entirely as we better understand the factors at play in this complex disease.

To date, only filaggrin deficiency, STAT3-HIES, DIDS, Netherton syndrome, and WAS have had their underlying cutaneous dysbiosis studied. However, the available literature regarding the cutaneous microbiomes of patients with WAS predominantly describes patients without the severe eczematous phenotype that is most characteristic of most patients with WAS (63). The other PADs reviewed in Table 1 (with the exception of filaggrin deficiency) have little known regarding the cutaneous microbiome changes which may or may not be unique to these disorders, and more studies replicating prior research and focused on patients with other PADs are clearly needed.

Unfortunately, PADs present a group of diseases that are exceptionally difficult to study due to small patient populations, generally young patients, extensive heterogeneity among patient presentations, and environmental factors, all of which lead to limitations in microbiome research findings. AD itself has high variability with age, as does the cutaneous microbiome, making research conducted on adult populations difficult to apply to most patients with PADs, which typically present and are diagnosed at a young age. Given the relative rarity of these patients, careful monitoring, documentation, and sample collection (when possible) will be crucial to facilitate future research.

Additionally, a number of potential biomarkers have been identified in recent years for earlier recognition of AD to facilitate more aggressive recognition and treatment. Stratum corneum lipids and certain cytokines have already been identified as early biomarkers for AD onset and severity (91, 92), yet few clinically viable microbial-derived biomarkers have been identified to date. Currently, there is strong evidence for early cutaneous microbiome changes as a risk factor for development of AD (93, 94), although utility for testing prior to AD-onset remains limited. Nasal and gut *S. aureus* colonization has been observed in patients with AD, but the clinical use of this measure is uncertain as *S. aureus* presence is ubiquitous in patients with AD and measures of *S. aureus* quantity and propensity for biofilm formation are limited (95, 96). Skin microbiota shifts have been repeatedly identified with AD treatment (39, 40, 44, 95, 97), suggesting a role for microbiome-based assays (either direct microbial population testing or measuring microbe-derived metabolites) as future biomarkers for treatment response. While this research remains in its infancy, future clinical application options will present additional diagnostic and monitoring parameters clinicians can utilize to help patients. Improved understanding of the differences noted in patients with PADs, such as colonization with unusual cutaneous microbes, may provide further diagnostic clues for an underlying PAD.

Patients with PADs continue to present clinical challenges for treating providers and understanding their unique traits may greatly impact treatment courses. With the advent and availability of advanced genetic testing, we anticipate more patients being identified, earlier recognition of disease, more targeted treatments (including bacteriotherapy, biologics, and small molecules), and improved outcomes for patients with PADs in the future.

## References

[1] Bantz SK Zhu Z Zheng T. The atopic march: progression from atopic dermatitis to allergic rhinitis and asthma. J Clin Cell Immunol. (2014) 5(2):202. 10.4172/2155-9899.1000202 25419479 PMC4240310

[2] Czarnowicki T Krueger JG Guttman-Yassky E. Novel concepts of prevention and treatment of atopic dermatitis through barrier and immune manipulations with implications for the atopic March. J Allergy Clin Immunol. (2017) 139(6):1723–34. 10.1016/j.jaci.2017.04.004 28583445

[3] Bawany F Beck LA Jarvinen KM. Halting the march: primary prevention of atopic dermatitis and food allergies. J Allergy Clin Immunol Pract. (2020) 8(3):860–75. 10.1016/j.jaip.2019.12.005 32147139 PMC7355223

[4] Lowe AJ Leung DYM Tang MLK Su JC Allen KJ. The skin as a target for prevention of the atopic March. Ann Allergy Asthma Immunol. (2018) 120(2):145–51. 10.1016/j.anai.2017.11.023 29413338

[5] Puerta Durango K Chiesa Fuxench ZC. Global burden of atopic dermatitis: examining disease prevalence across pediatric and adult populations world-wide. Dermatol Clin. (2024) 42(4):519–25. 10.1016/j.det.2024.05.004 39278705

[6] Demessant-Flavigny AL Connetable S Kerob D Moreau M Aguilar L Wollenberg A. Skin microbiome dysbiosis and the role of *Staphylococcus Aureus* in atopic dermatitis in adults and children: a narrative review. J Eur Acad Dermatol Venereol. (2023) 37(Suppl 5):3–17. 10.1111/jdv.19125 37232427

[7] Baurecht H Ruhlemann MC Rodriguez E Thielking F Harder I Erkens AS Epidermal lipid composition, barrier integrity, and eczematous inflammation are associated with skin microbiome configuration. J Allergy Clin Immunol. (2018) 141(5):1668–76.e16. 10.1016/j.jaci.2018.01.019 29421277

[8] Cau L Williams MR Butcher AM Nakatsuji T Kavanaugh JS Cheng JY *Staphylococcus Epidermidis* protease ecpa can be a deleterious component of the skin microbiome in atopic dermatitis. J Allergy Clin Immunol. (2021) 147(3):955–66.e16. 10.1016/j.jaci.2020.06.024 32634452 PMC8058862

[9] Nakatsuji T Chen TH Narala S Chun KA Two AM Yun T Antimicrobials from human skin commensal Bacteria protect against *Staphylococcus Aureus* and are deficient in atopic dermatitis. Sci Transl Med. (2017) 9(378):eaah4680. 10.1126/scitranslmed.aah4680 28228596 PMC5600545

[10] Glatthardt T Lima RD de Mattos RM Ferreira RBR. Microbe interactions within the skin microbiome. Antibiotics (Basel). (2024) 13(1):49. 10.3390/antibiotics13010049 38247608 PMC10812674

[11] Cogen AL Yamasaki K Muto J Sanchez KM Crotty Alexander L Tanios J *Staphylococcus Epidermidis* antimicrobial Delta-toxin (phenol-soluble modulin-gamma) cooperates with host antimicrobial peptides to kill group a Streptococcus. PLoS One. (2010) 5(1):e8557. 10.1371/journal.pone.0008557 20052280 PMC2796718

[12] Traisaeng S Herr DR Kao HJ Chuang TH Huang CM. A derivative of butyric acid, the fermentation metabolite of *Staphylococcus Epidermidis*, inhibits the growth of a *Staphylococcus Aureus* strain isolated from atopic dermatitis patients. Toxins (Basel). (2019) 11(6):311. 10.3390/toxins11060311 31159213 PMC6628397

[13] Glatthardt T Mello Campos JC Chamon RC Sá Coimbra TF Almeida Rocha G Melo MAF Small molecules produced by commensal *Staphylococcus Epidermidis* disrupt formation of biofilms by *Staphylococcus Aureus*. Appl Environ Microbiol. (2020) 86(5):e02539–19. 10.1128/AEM 31862721 PMC7028967

[14] Zheng Y Hunt RL Villaruz AE Fisher EL Liu R Liu Q Commensal *Staphylococcus Epidermidis* contributes to skin barrier homeostasis by generating protective ceramides. Cell Host Microbe. (2022) 30(3):301–13.e9. 10.1016/j.chom.2022.01.004 35123653 PMC8917079

[15] Lai Y Cogen AL Radek KA Park HJ Macleod DT Leichtle A Activation of Tlr2 by a small molecule produced by *Staphylococcus Epidermidis* increases antimicrobial defense against bacterial skin infections. J Invest Dermatol. (2010) 130(9):2211–21. 10.1038/jid.2010.123 20463690 PMC2922455

[16] Mahmud MR Akter S Tamanna SK Mazumder L Esti IZ Banerjee S Impact of gut microbiome on skin health: gut-skin axis observed through the lenses of therapeutics and skin diseases. Gut Microbes. (2022) 14(1):2096995. 10.1080/19490976.2022.2096995 35866234 PMC9311318

[17] Hazime R Eddehbi FE El Mojadili S Lakhouaja N Souli I Salami A Inborn errors of immunity and related microbiome. Front Immunol. (2022) 13:982772. 10.3389/fimmu.2022.982772 36177048 PMC9513548

[18] Luger T Amagai M Dreno B Dagnelie MA Liao W Kabashima K Atopic dermatitis: role of the skin barrier, environment, microbiome, and therapeutic agents. J Dermatol Sci. (2021) 102(3):142–57. 10.1016/j.jdermsci.2021.04.007 34116898

[19] Hoskinson C Medeleanu MV Reyna ME Dai DLY Chowdhury B Moraes TJ Antibiotics taken within the first year of life are linked to infant gut microbiome disruption and elevated atopic dermatitis risk. J Allergy Clin Immunol. (2024) 154(1):131–42. 10.1016/j.jaci.2024.03.025 38670232

[20] Prescott SL Larcombe DL Logan AC West C Burks W Caraballo L The skin microbiome: impact of modern environments on skin ecology, barrier integrity, and systemic immune programming. World Allergy Organ J. (2017) 10(1):29. 10.1186/s40413-017-0160-5 28855974 PMC5568566

[21] Tham EH Chia M Riggioni C Nagarajan N Common JEA Kong HH. The skin microbiome in pediatric atopic dermatitis and food allergy. Allergy. (2024) 79(6):1470–84. 10.1111/all.16044 38308490 PMC11142881

[22] Chong AC Visitsunthorn K Ong PY. Genetic/environmental contributions and immune dysregulation in children with atopic dermatitis. J Asthma Allergy. (2022) 15:1681–700. 10.2147/JAA.S293900 36447957 PMC9701514

[23] Vaseghi-Shanjani M Samra S Yousefi P Biggs CM Turvey SE. Primary atopic disorders: inborn errors of immunity causing severe allergic disease. Curr Opin Immunol. (2025) 94:102538. 10.1016/j.coi.2025.102538 40020536

[24] Vaseghi-Shanjani M Smith KL Sara RJ Modi BP Branch A Sharma M Inborn errors of immunity manifesting as atopic disorders. J Allergy Clin Immunol. (2021) 148(5):1130–9. 10.1016/j.jaci.2021.08.008 34428518

[25] Castagnoli R Lougaris V Giardino G Volpi S Leonardi L La Torre F Inborn errors of immunity with atopic phenotypes: a practical guide for allergists. World Allergy Organ J. (2021) 14(2):100513. 10.1016/j.waojou.2021.100513 33717395 PMC7907539

[26] Cinicola BL Corrente S Castagnoli R Lougaris V Giardino G Leonardi L Primary atopic disorders and chronic skin disease. Pediatr Allergy Immunol. (2022) 33(Suppl 27):65–8. 10.1111/pai.13633 35080318 PMC9306837

[27] Sacco KA Milner JD. Gene-environment interactions in primary atopic disorders. Curr Opin Immunol. (2019) 60:148–55. 10.1016/j.coi.2019.06.002 31302571

[28] Pellicciotta M Rigoni R Falcone EL Holland SM Villa A Cassani B. The microbiome and immunodeficiencies: lessons from rare diseases. J Autoimmun. (2019) 98:132–48. 10.1016/j.jaut.2019.01.008 30704941

[29] Ozdemir O. Relation between dysbiosis and inborn errors of immunity. World J Methodol. (2024) 14(4):96380. 10.5662/wjm.v14.i4.96380 39712559 PMC11287548

[30] Edslev SM Agner T Andersen PS. Skin microbiome in atopic dermatitis. Acta Derm Venereol. (2020) 100(12):adv00164. 10.2340/00015555-3514 32419029 PMC9189751

[31] Han SH Cheon HI Hur MS Kim MJ Jung WH Lee YW Analysis of the skin mycobiome in adult patients with atopic dermatitis. Exp Dermatol. (2018) 27(4):366–73. 10.1111/exd.13500 29356103

[32] Bjerre RD Holm JB Palleja A Solberg J Skov L Johansen JD. Skin dysbiosis in the microbiome in atopic dermatitis is site-specific and involves Bacteria, fungus and virus. BMC Microbiol. (2021) 21(1):256. 10.1186/s12866-021-02302-2 34551705 PMC8459459

[33] Glatz M Jo JH Kennedy EA Polley EC Segre JA Simpson EL Emollient use alters skin barrier and microbes in infants at risk for developing atopic dermatitis. PLoS One. (2018) 13(2):e0192443. 10.1371/journal.pone.0192443 29489859 PMC5830298

[34] Byrd AL Deming C Cassidy SKB Harrison OJ Ng WI Conlan S *Staphylococcus Aureus* and *Staphylococcus Epidermidis* strain diversity underlying pediatric atopic dermatitis. Sci Transl Med. (2017) 9(397):eaal4651. 10.1126/scitranslmed.aal4651 28679656 PMC5706545

[35] Chng KR Tay AS Li C Ng AH Wang J Suri BK Whole metagenome profiling reveals skin microbiome-dependent susceptibility to atopic dermatitis flare. Nat Microbiol. (2016) 1(9):16106. 10.1038/nmicrobiol.2016.106 27562258

[36] Salava A Lauerma A. Role of the skin microbiome in atopic dermatitis. Clin Transl Allergy. (2014) 4:33. 10.1186/2045-7022-4-33 25905004 PMC4405870

[37] Yu SH Lio P. External factors and the cutaneous microbiome. J Integr Dermatol. (2024) 1(1). 10.64550/joid.hjrcmt43

[38] Callewaert C Ravard Helffer K Lebaron P. Skin microbiome and its interplay with the environment. Am J Clin Dermatol. (2020) 21(Suppl 1):4–11. 10.1007/s40257-020-00551-x 32910439 PMC7584520

[39] Huang C Zhuo F Guo Y Wang S Zhang K Li X Skin microbiota: pathogenic roles and implications in atopic dermatitis. Front Cell Infect Microbiol. (2024) 14:1518811. 10.3389/fcimb.2024.1518811 39877655 PMC11772334

[40] Kong HH Oh J Deming C Conlan S Grice EA Beatson MA Temporal shifts in the skin microbiome associated with disease flares and treatment in children with atopic dermatitis. Genome Res. (2012) 22(5):850–9. 10.1101/gr.131029.111 22310478 PMC3337431

[41] Gonzalez ME Schaffer JV Orlow SJ Gao Z Li H Alekseyenko AV Cutaneous microbiome effects of fluticasone propionate cream and adjunctive bleach baths in childhood atopic dermatitis. J Am Acad Dermatol. (2016) 75(3):481–93.e8. 10.1016/j.jaad.2016.04.066 27543211 PMC4992571

[42] Wongpiyabovorn J Soonthornchai W Wilantho A Palasuk M Payungporn S Sodsai P Effect of tacrolimus on skin microbiome in atopic dermatitis. Allergy. (2019) 74(7):1400–6. 10.1111/all.13743 30742708

[43] Callewaert C Nakatsuji T Knight R Kosciolek T Vrbanac A Kotol P Il-4ralpha blockade by dupilumab decreases *Staphylococcus Aureus* colonization and increases microbial diversity in atopic dermatitis. J Invest Dermatol. (2020) 140(1):191–202.e7. 10.1016/j.jid.2019.05.024 31252032 PMC7163930

[44] Olesen CM Ingham AC Thomsen SF Clausen ML Andersen PS Edslev SM Changes in skin and nasal microbiome and staphylococcal species following treatment of atopic dermatitis with dupilumab. Microorganisms. (2021) 9(7):1487. 10.3390/microorganisms9071487 34361924 PMC8303790

[45] Simpson EL Schlievert PM Yoshida T Lussier S Boguniewicz M Hata T Rapid reduction in *Staphylococcus Aureus* in atopic dermatitis subjects following dupilumab treatment. J Allergy Clin Immunol. (2023) 152(5):1179–95. 10.1016/j.jaci.2023.05.026 37315812 PMC10716365

[46] Beck LA Bieber T Weidinger S Tauber M Saeki H Irvine AD Tralokinumab treatment improves the skin Microbiota by increasing the microbial diversity in adults with moderate-to-severe atopic dermatitis: analysis of microbial diversity in ecztra 1, a randomized controlled trial. J Am Acad Dermatol. (2023) 88(4):816–23. 10.1016/j.jaad.2022.11.047 36473633

[47] Stefanovic N Irvine AD. Filaggrin and beyond: new insights into the skin barrier in atopic dermatitis and allergic diseases, from genetics to therapeutic perspectives. Ann Allergy Asthma Immunol. (2024) 132(2):187–95. 10.1016/j.anai.2023.09.009 37758055

[48] Prajapati SK Lekkala L Yadav D Jain S Yadav H. Microbiome and postbiotics in skin health. Biomedicines. (2025) 13(4):791. 10.3390/biomedicines13040791 40299368 PMC12025169

[49] Bellanti JA. Selected inborn errors of immunity associated with severe atopic phenotypes: implications for the practicing allergist. Ann Allergy Asthma Immunol. (2025) 135(2):162–8. 10.1016/j.anai.2025.05.024 40449791

[50] Smieszek SP Welsh S Xiao C Wang J Polymeropoulos C Birznieks G Correlation of age-of-onset of atopic dermatitis with filaggrin loss-of-function variant status. Sci Rep. (2020) 10(1):2721. 10.1038/s41598-020-59627-7 32066784 PMC7026049

[51] Weidinger S Illig T Baurecht H Irvine AD Rodriguez E Diaz-Lacava A Loss-of-function variations within the filaggrin gene predispose for atopic dermatitis with allergic sensitizations. J Allergy Clin Immunol. (2006) 118(1):214–9. 10.1016/j.jaci.2006.05.004 16815158

[52] Sandilands A Sutherland C Irvine AD McLean WH. Filaggrin in the frontline: role in skin barrier function and disease. J Cell Sci. (2009) 122(Pt 9):1285–94. 10.1242/jcs.033969 19386895 PMC2721001

[53] Smith FJ Irvine AD Terron-Kwiatkowski A Sandilands A Campbell LE Zhao Y Loss-of-function mutations in the gene encoding filaggrin cause ichthyosis Vulgaris. Nat Genet. (2006) 38(3):337–42. 10.1038/ng1743 16444271

[54] Margolis DJ Mitra N Wubbenhorst B Nathanson KL. Filaggrin sequencing and bioinformatics tools. Arch Dermatol Res. (2020) ) 312(2):155–8. 10.1007/s00403-019-01956-3 31372728 PMC6994326

[55] Clausen ML Agner T Lilje B Edslev SM Johannesen TB Andersen PS. Association of disease severity with skin microbiome and filaggrin gene mutations in adult atopic dermatitis. JAMA Dermatol. (2018) 154(3):293–300. 10.1001/jamadermatol.2017.5440 29344612 PMC5885821

[56] Patrick GJ Liu H Alphonse MP Dikeman DA Youn C Otterson JC Epicutaneous *Staphylococcus Aureus* induces il-36 to enhance IgE production and ensuing allergic disease. J Clin Invest. (2021) 131(5):e143334. 10.1172/JCI143334 33645549 PMC7919715

[57] Levy DE Lee C-K. What does Stat3 do? J Clin Invest. (2002) 109(9):1143–8. 10.1172/jci0215650 11994402 PMC150972

[58] Kasap N Kara A Celik V Bilgic Eltan S Akay Haci I Kose H Atypical localization of eczema discriminates Dock8 or Stat3 deficiencies from atopic dermatitis. J Clin Immunol. (2023) 43(8):1882–90. 10.1007/s10875-023-01554-z 37507632

[59] Hoskins S Skoda-Smith S Torgerson TR Boos MD. Eczematous dermatitis in primary immunodeficiencies: a review of cutaneous clues to diagnosis. Clin Immunol. (2020) 211:108330. 10.1016/j.clim.2019.108330 31899331

[60] James AE West L Schloss K Nataraj P Urban A Hirsch A Treatment of Stat3-deficient hyper-immunoglobulin E syndrome with monoclonal antibodies targeting allergic inflammation. J Allergy Clin Immunol Pract. (2022) 10(5):1367–70.e1. 10.1016/j.jaip.2022.01.011 35085810

[61] Dick JK Boull C Pozos TC Maguiness SM. Improvement in atopic dermatitis and recurrent infection with dupilumab in children with distinct genetic types of hyper-IgE syndrome: a case series and literature review. Pediatr Dermatol. (2025) 42(2):376–82. 10.1111/pde.15780 39420803 PMC11950812

[62] Olbrich H Sadik CD Ludwig RJ Thaci D Boch K. Dupilumab in inflammatory skin diseases: a systematic review. Biomolecules. (2023) 13(4):634. 10.3390/biom13040634 37189381 PMC10136243

[63] Oh J Freeman AF Program NCS Park M Sokolic R Candotti F The altered landscape of the human skin microbiome in patients with primary immunodeficiencies. Genome Res. (2013) 23(12):2103–14. 10.1101/gr.159467.113 24170601 PMC3847779

[64] Sastalla I Williams KW Anderson ED Myles IA Reckhow JD Espinoza-Moraga M Molecular typing of *Staphylococcus Aureus* isolated from patients with autosomal dominant hyper IgE syndrome. Pathogens. (2017) 6(2):23. 10.3390/pathogens6020023 28587312 PMC5488657

[65] Smeekens SP Huttenhower C Riza A van de Veerdonk FL Zeeuwen PL Schalkwijk J Skin microbiome imbalance in patients with Stat1/Stat3 defects impairs innate host defense responses. J Innate Immun. (2014) 6(3):253–62. 10.1159/000351912 23796786 PMC4045018

[66] Fusaro M Dupre L. Mechanisms underlying skin inflammation of Dock8 deficiency. J Allergy Clin Immunol. (2024) 154(1):88–90. 10.1016/j.jaci.2024.04.026 38759801

[67] Gray PE David C. Inborn errors of immunity and autoimmune disease. J Allergy Clin Immunol Pract. (2023) 11(6):1602–22. 10.1016/j.jaip.2023.04.018 37119983

[68] Tirosh O Conlan S Deming C Lee-Lin SQ Huang X Program NCS Expanded skin virome in Dock8-deficient patients. Nat Med. (2018) 24(12):1815–21. 10.1038/s41591-018-0211-7 30397357 PMC6286253

[69] Anderson M Newell B Escobar H Stahl E Raje N. A case of Dock8 deficiency treated with dupilumab. Ann Allergy Asthma Immunol. (2022) 129(5):S141. 10.1016/j.anai.2022.08.912

[70] Che Y Han J Harkins CP Hou P Conlan S Deming C Restoration of the human skin microbiome following immune recovery after hematopoietic stem cell transplantation. Cell Host Microbe. (2025) 33(8):1412–27.e5. 10.1016/j.chom.2025.07.002 40730159 PMC12313297

[71] Spencer S Kostel Bal S Egner W Lango Allen H Raza SI Ma CA Loss of the interleukin-6 receptor causes immunodeficiency, atopy, and abnormal inflammatory responses. J Exp Med. (2019) 216(9):1986–98. 10.1084/jem.20190344 31235509 PMC6719421

[72] Schwerd T Twigg SRF Aschenbrenner D Manrique S Miller KA Taylor IB A biallelic mutation in Il6st encoding the Gp130 co-receptor causes immunodeficiency and craniosynostosis. J Exp Med. (2017) 214(9):2547–62. 10.1084/jem.20161810 28747427 PMC5584118

[73] Beziat V Tavernier SJ Chen YH Ma CS Materna M Laurence A Dominant-negative mutations in human Il6st underlie hyper-IgE syndrome. J Exp Med. (2020) 217(6):e20191804. 10.1084/jem.20191804 32207811 PMC7971136

[74] Minskaia E Maimaris J Jenkins P Albuquerque AS Hong Y Eleftheriou D Autosomal dominant Stat6 gain of function causes severe atopy associated with lymphoma. J Clin Immunol. (2023) 43(7):1611–22. 10.1007/s10875-023-01530-7 37316763 PMC10499697

[75] James AE Abdalgani M Khoury P Freeman AF Milner JD. T(H)2-driven manifestations of inborn errors of immunity. J Allergy Clin Immunol. (2024) 154(2):245–54. 10.1016/j.jaci.2024.05.007 38761995 PMC12295673

[76] Pichard DC Freeman AF Cowen EW. Primary immunodeficiency update: part I. Syndromes associated with eczematous dermatitis. J Am Acad Dermatol. (2015) 73(3):355–64; quiz 65–6. 10.1016/j.jaad.2015.01.054 26282794 PMC4542006

[77] Albert MH Freeman AF. Wiskott-Aldrich syndrome (was) and dedicator of cytokinesis 8- (Dock8) deficiency. Front Pediatr. (2019) 7:451. 10.3389/fped.2019.00451 31750279 PMC6848221

[78] Maillard MH Cotta-de-Almeida V Takeshima F Nguyen DD Michetti P Nagler C The Wiskott-Aldrich syndrome protein is required for the function of Cd4(+)Cd25(+)Foxp3(+) regulatory T cells. J Exp Med. (2007) 204(2):381–91. 10.1084/jem.20061338 17296786 PMC2118715

[79] Herman KE Yoshida T Hughson A Grier A Gill SR Beck LA Il-17-Dependent dysregulated cutaneous immune homeostasis in the absence of the Wiskott-Aldrich syndrome protein. Front Immunol. (2022) 13:817427. 10.3389/fimmu.2022.817427 35265075 PMC8900519

[80] Boztug K Schmidt M Schwarzer A Banerjee PP Avedillo Díez I Dewey RA Stem-cell gene therapy for the Wiskott–Aldrich syndrome. N Engl J Med. (2010) 363(20):1918–27. 10.1056/NEJMoa1003548 21067383 PMC3064520

[81] Mallhi KK Petrovic A Ochs HD. Hematopoietic stem cell therapy for Wiskott-Aldrich syndrome: improved outcome and quality of life. J Blood Med. (2021) 12:435–47. 10.2147/JBM.S232650 34149291 PMC8206065

[82] Dorjbal B Stinson JR Ma CA Weinreich MA Miraghazadeh B Hartberger JM Hypomorphic caspase activation and recruitment domain 11 (Card11) mutations associated with diverse immunologic phenotypes with or without atopic disease. J Allergy Clin Immunol. (2019) 143(4):1482–95. 10.1016/j.jaci.2018.08.013 30170123 PMC6395549

[83] Diaz-Cabrera NM Bauman BM Iro MA Dabbah-Krancher G Molho-Pessach V Zlotogorski A Management of atopy with dupilumab and omalizumab in cadins disease. J Clin Immunol. (2024) 44(2):48. 10.1007/s10875-023-01636-y 38231347

[84] Punwani D Wang H Chan AY Cowan MJ Mallott J Sunderam U Combined immunodeficiency due to Malt1 mutations, treated by hematopoietic cell transplantation. J Clin Immunol. (2015) 35(2):135–46. 10.1007/s10875-014-0125-1 25627829 PMC4352191

[85] Rozmus J McDonald R Fung SY Del Bel KL Roden J Senger C Successful clinical treatment and functional immunological normalization of human Malt1 deficiency following hematopoietic stem cell transplantation. Clin Immunol. (2016) 168:1–5. 10.1016/j.clim.2016.04.011 27109639

[86] Williams MR Cau L Wang Y Kaul D Sanford JA Zaramela LS Interplay of staphylococcal and host proteases promotes skin barrier disruption in Netherton syndrome. Cell Rep. (2020) 30(9):2923–33.e7. 10.1016/j.celrep.2020.02.021 32130897 PMC7183042

[87] Sillanpaa V Soratto TAT Eranko E Barrientos-Somarribas M Hannula-Jouppi K Andersson B Skin microbiota and clinical associations in Netherton syndrome. JID Innov. (2021) 1(2):100008. 10.1016/j.xjidi.2021.100008 34909712 PMC8659401

[88] Stuvel K Heeringa JJ Dalm V Meijers RWJ van Hoffen E Gerritsen SAM Comel-Netherton syndrome: a local skin barrier defect in the absence of an underlying systemic immunodeficiency. Allergy. (2020) 75(7):1710–20. 10.1111/all.14197 31975472 PMC7384150

[89] Nelson RW Geha RS McDonald DR. Inborn errors of the immune system associated with atopy. Front Immunol. (2022) 13:860821. 10.3389/fimmu.2022.860821 35572516 PMC9094424

[90] Blaustein RA Shen Z Kashaf SS Lee-Lin S Conlan S Program NCS Expanded microbiome niches of rag-deficient patients. Cell Rep Med. (2023) 4(10):101205. 10.1016/j.xcrm.2023.101205 37757827 PMC10591041

[91] Berdyshev E Kim J Kim BE Goleva E Lyubchenko T Bronova I Stratum corneum lipid and cytokine biomarkers at age 2 months predict the future onset of atopic dermatitis. J Allergy Clin Immunol. (2023) 151(5):1307–16. 10.1016/j.jaci.2023.02.013 36828081

[92] Kim HB Alexander H Um JY Chung BY Park CW Flohr C Skin microbiome dynamics in atopic dermatitis: understanding host-microbiome interactions. Allergy Asthma Immunol Res. (2025) 17(2):165–80. 10.4168/aair.2025.17.2.165 40204503 PMC11982640

[93] Kennedy EA Connolly J Hourihane JO Fallon PG McLean WHI Murray D Skin microbiome before development of atopic dermatitis: early colonization with commensal staphylococci at 2 months is associated with a lower risk of atopic dermatitis at 1 year. J Allergy Clin Immunol. (2017) 139(1):166–72. 10.1016/j.jaci.2016.07.029 27609659 PMC5207796

[94] Meylan P Lang C Mermoud S Johannsen A Norrenberg S Hohl D Skin colonization by *Staphylococcus Aureus* precedes the clinical diagnosis of atopic dermatitis in infancy. J Invest Dermatol. (2017) 137(12):2497–504. 10.1016/j.jid.2017.07.834 28842320

[95] Augusto de Oliveira MF Agne DB Bastos LSS Andrade de Oliveira LM Saintive S Goudouris ES Atopic dermatitis pediatric patients show high rates of nasal and intestinal colonization by methicillin-resistant *Staphylococcus Aureus* and coagulase-negative staphylococci. BMC Microbiol. (2024) 24(1):42. 10.1186/s12866-023-03165-5 38287251 PMC10823624

[96] Guo Y Dou X Chen XF Huang C Zheng YJ Yu B. Association between nasal colonization of *Staphylococcus Aureus* and eczema of multiple body sites. Allergy Asthma Immunol Res. (2023) 15(5):659–72. 10.4168/aair.2023.15.5.659 37827982 PMC10570784

[97] Reiger M Traidl-Hoffmann C Neumann AU. The skin microbiome as a clinical biomarker in atopic eczema: promises, navigation, and pitfalls. J Allergy Clin Immunol. (2020) 145(1):93–6. 10.1016/j.jaci.2019.11.004 31910987

